# Bioimpedance spectroscopy for swelling evaluation following total knee arthroplasty: a validation study

**DOI:** 10.1186/s12891-015-0559-5

**Published:** 2015-04-25

**Authors:** Claude Pichonnaz, Jean-Philippe Bassin, Estelle Lécureux, Damien Currat, Brigitte M Jolles

**Affiliations:** Physiotherapy Department, Haute Ecole de Santé Vaud (HESAV), HES-SO, University of Applied Sciences Western Switzerland, Delémont, Switzerland; CHUV-UNIL, Orthopedics and Traumatology Department, CHUV-UNIL, Avenue du Bugnon 21, 1011 Lausanne, Switzerland; CHUV-UNIL, direction médicale, Rue du Bugnon 46, 1011 Lausanne, Switzerland

**Keywords:** Validation study, Reproducibility of results, ROC curve, Edema, Arthroplasty, Replacement (MeSH terms)

## Abstract

**Background:**

The evaluation of swelling is important for the outcome of total knee arthroplasty (TKA) surgery. The circumference or volume measurements are applicable at the bedside of the patient but are altered by muscular atrophy and the post-surgical dressing. Bioimpedance spectroscopy might overcome these limitations; however, it should be validated. This study aimed to explore the validity, the reliability and the responsiveness of bioimpedance spectroscopy for measuring swelling after TKA.

**Methods:**

The degree of swelling in 25 patients undergoing TKA surgery was measured using bioimpedance spectroscopy (BIS R0), knee circumference and limb volume. The measurements were performed on D-1 (day before surgery), D + 2 (2 days after surgery) and D + 8 (8 days after surgery). The BIS R0 measurements were repeated twice, alternating between two evaluators. The percentage of the difference between the limbs was calculated for BIS R0, circumference and volume. The intra- and inter-observer intraclass correlation coefficients (ICCs), limits of agreement (LOA), effect size (Cohen’s *d*), correlations between the methods and diagnostic sensitivity were calculated.

**Results:**

BIS R0, circumference and volume detected swelling < 3.5% at D-1. The swelling at D2 and D8 was greater with BIS R0 [mean (SD) 29.9% (±9.8) and 38.27 (±7.8)] than with volume [14.7 (±9.5) and 14.9 (±8.2)] and circumference [11.1 (±5.7) and 11.7 (±4.1)].

The BIS R0 intra- and inter-evaluator ICCs ranged from 0.89 to 0.99, whereas the LOA were < 5.2%. The BIS R0 correlation was 0.73 with volume and 0.75 with circumference. The BIS R0 Cohen’s *d* was 3.32 for the D-1–D2 evolution. The diagnostic sensitivity was 83% D2 and 96% at D8.

**Conclusion:**

Bioimpedance is a valid method for the evaluation of swelling following TKA. BIS R0 also demonstrated excellent intra- and inter- evaluator reliability. The diagnostic sensitivity and responsiveness is superior to that of concurrent methods. BIS R0 is an efficient method for post-surgical follow up at the bedside of the patient. The measurement of BIS R0 is a straightforward, valid, reliable and responsive method for lower limb swelling following TKA surgery that could be used in clinics and research.

**Trial registration:**

ClinicalTrials.gov Identifier: NCT00627770.

## Background

Total knee arthroplasty (TKA) is a very common knee surgical intervention the frequency of which will increase markedly in the future [1]. It is important to improve post-surgical swelling follow up methods to optimize the quality of care and control TKA-related health costs. Several authors have suggested that swelling influences the post-surgical evolution and patient satisfaction [2-7]. Swelling has been related to increased pain [8], decreased range of motion of the knee [9], gait alteration [10], decreased quadriceps strength [11,12] and delayed recovery [13].

The effects of limb position [14,15], lymphatic drainage [16], magnetic pulsed field [17,18], cryotherapy [19,20] compression stockings [21], electrostimulation [22] and pumps [23] have been investigated. All of the cited studies used knee tape girth measurement as the measurement outcome. Although straightforward and reliable, limb circumference measurement presents several disadvantages in this context [24]. It is influenced by variations of muscular mass whereas atrophy might be consequent following surgery [25]. Swelling might be underestimated following TKA surgery and its application might be complicated by the post-surgical volume evaluation determined by tape measurements at regular intervals is also a valid and reliable measurement method [26-29]. It provides a more extensive measurement of the lower limbs but is relatively time-consuming.

Other possible measurement methods (imaging technology, optometric measurement and water displacement) are hardly applicable in routine post-surgical management because of issues of convenience and cost [30-32]. A valid, reliable, responsive and straightforward, post-surgical swelling evaluation method applicable at the patient bedside would facilitate the evaluation of swelling for clinicians and researchers. This could thus contribute to the development of knowledge about the causes, consequences and treatment of swelling from the early post-surgical stage.

Electrical bioimpedance measurement might meet these specifications. Normal bioimpedance devices are portable, the measurement time is limited to the placement of the electrodes and the result is immediate. The basic principle of electrical bioimpedance consists of analyzing the alterations of an imperceptible alternating current (200 mA) administered through electrodes across the body or body segment. The body composition could be determined based on the opposition of living tissues (impedance) to this current [33]. Because the opposition to the electrical current decreases when the fluid volume increases, the limb impedance drops in cases of swelling. Bioimpedance can thus be applied, among other medical applications, to assess limb edema and monitor its evolution [33].

The limb extracellular fluid (ECF) is measured by the R0 (R-zero) variable is of major interest for the evaluation of limb edema [33,34]. R0 refers to the resistance to an electrical current at a frequency theoretically equal to zero, as with a continuous direct current. This variable is straightforwardly measured using a bioimpedance spectroscopy (BIS) device.

Following TKA surgery, R0 is not altered by the arthroplasty metallic implant [35]. As the current flows primarily through the extracellular compartment, it reflects solely the ECF in which most edema occurs. Post-surgical swelling is constituted by edema, hematomas and joint effusion, of which ECF is accounted for in the BIS R0 measurement. R0 could thus be considered to be a potentially valid estimator of post-surgical swelling. BIS R0 is highly correlated to volume (determined by perometry, water displacement or tape measurement) and demonstrates high diagnostic power for the detection of lymphedema [36-41].

However, this assumption must be confirmed by further research because, to our knowledge, this measurement method has not been previously applied following surgery.

Despite the practical advantage and established measurement properties of BIS limb ratio measurement, its validity must be investigated following TKA surgery. Additionally, the intra- and inter-evaluator reproducibility, responsiveness and relationship to volume measurement must be extensively investigated before its application in the evaluation of post-surgical lower limb swelling. The aim of this study was to investigate extensively the BIS R0 variable measurement properties for the evaluation of swelling following TKA surgery.

## Methods

This prospective validation study was conducted in the Department of Orthopedic and Traumatic Surgery of the University Hospital of Lausanne. Ethical approval was granted by the local ethics committee (Cantonal commission for Ethics for the Research on Human Beings of the University of Lausanne # 135/07). Written informed consent for participation in the study was obtained from participants.

A group of 25 patients who had undergone TKA surgery was included. The sample size was determined along a probability of type 2 error of 0.05, a power of 0.8, a coefficient of correlation of at least moderate strength (r = 0.50) and accounting for a patient dropout rate of 15%.

The patients who were operated on for a primary TKA for osteoarthritis according to the standard surgical procedure of the department were included. They were enrolled one after another on admission, provided that two evaluators were available to conduct the measurements. The exclusion criteria were a lower limb metallic implant other than a TKA, a pre-existing edema, a pacemaker or a cardiac defibrillator.

The BIS and volume measures were taken the day before surgery (baseline), two days after surgery (D2) and eight days after surgery (D8). Pairs of evaluators performed the measurements. The evaluators’ order was randomly determined by throwing a coin. The first evaluator only initially performed the limb volume measurements to ensure that the patient had been lying down for at least 10 minutes before the bioimpedance measurement. The BIS R0 measurements were then repeated twice with an Impedimed SFB7 device (Pinkenba, QLD, Australia), alternating between the two evaluators. The percentage difference between the healthy and the involved limb were respectively calculated for volume and for BIS R0.

The limb volume was determined using tape measurements at 4-cm intervals starting from the patella + 1 cm level. The volume was calculated using the truncated cone method [26]. Meijer et al. demonstrated that this method displayed a non-significant difference and excellent correlations with the volume determined by water displacement (r = 0.86 – 0.87), as well as excellent intra-evaluator reliability (Intraclass Correlation Coefficient (ICC) 0.90 to 0.99) and good inter-evaluator reliability (ICC 0.85 to 0.88) [28]. A no-stretch Gulick II tape (FitnessMartW, Gays Mills, WI, USA) was used. The most proximal measurement was performed at the highest level of the thigh at which it was possible not to skew the tape. The most distal measurement was the lowest one that could be taken above the largest cross-sectional area of the ankle. Based on preliminary measurements on healthy subjects with and without the standard post-surgical dressing, a deduction of 0.57 mm was applied on each tape measurement made on the dressing. Using this compensation, limb volume could be retrieved with a mean (SD) error of 0.1% (±0.25%).

A four-wire measurement method was used for the BIS measurements [35]. The most proximal electrode was placed on the level of the most proximal tape measurement, and the most distal electrode at the level of the most distal tape measurement. Thus the identical area was covered by the two concurrent measurement methods. The measurements were performed out of sight of the concurrent evaluator, the electrodes were taken off between measurements and new electrodes were used each time.

Swelling on the involved side was expressed as a positive percentage difference for all of the measurement methods. No correction for side dominance was used because dominance has a negligible influence on the lower limb swelling measurement [42,43].

The mean and standard deviation (SD) of the percentage difference between the limbs were calculated for BIS R0, volume and circumference at the patella + 1 cm level at all stages. The Wilcoxon signed rank test was used for the significance of the difference between the stages calculation. ANOVA was used for the difference between the evaluators and between the measurements by the same evaluator at all stages. The ICCs were calculated for the intra- and inter-evaluator relationship, as well as for the standard errors of measurement and the limits of agreement. An ICC below 0.75 was considered as poor to moderate, 0.75 to 0.90 as good, and above 0.90 as excellent reliability [43]. The effect size was calculated using the Cohen’s *d*. Spearman coefficients of correlation were used to establish the relationships between R0, volume and circumference. The sensitivity, the specificity and the optimal threshold to discriminate the pre- from the post-surgical swelling states were assessed using the receiver operating characteristic curve (ROC). The optimal threshold was defined as the one presenting the best balance between specificity and sensitivity. The significance level was set at P < 0.05 where applicable. The statistical analysis was performed using the SPSS statistical software, version 18 (SPSS, Chicago, Illinois) and STATA 11 (StataCorp, College Station, Texas).

### Sources of funding

This study was supported by the HES-SO (HESAV, HEIG-VD and Strategic Fund) and the Swiss National Physiotherapy Association Physioswiss. Funding sources supported the financial costs of the study, but did not play any role in the study’s design, conduct, or reporting.

## Results

Twenty-five patients were included in the study. One patient was excluded because of post-surgical thrombosis. The characteristics of the included patients are described in Table 1.

**Table 1:** **Patients’ characteristics**

**Patients’ characteristics**					
**Age, y mean (SD)**	**Weight, kg mean (SD)**	**Size, m mean (SD)**	**BMI, kg/m** ^**2**^ **mean (SD)**	**Female, n (%)**	**Arthroplasty right side, n (%)**
69.5 (9.7)	85.0 (14.3)	167.9 (4.8)	30.7 (4.8)	12 (50)	15 (62)

Legends = BMI: Body Mass Index; SD = Standard Deviation.

**Figure 1:**
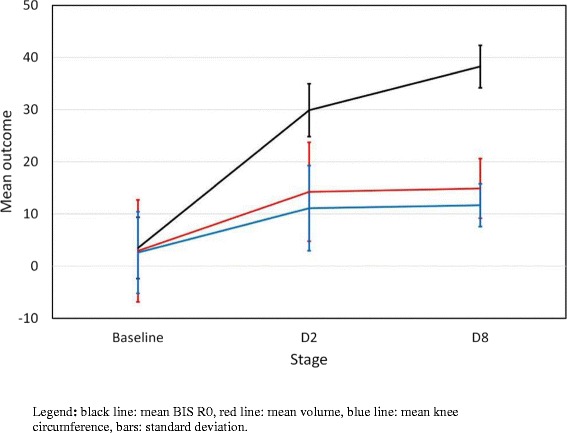
Comparison of swelling evolution according to the measurement method. Legend: black line: mean BIS R0, red’ line: mean volume, blue line: mean knee circumference, bars: standard deviation.

The mean (SD) percentage difference between the limbs at all stages for volume, circumference and bioimpedance are reported in Table 2 and Figure 1. Significant differences were found between the baseline and D2 for all of the measurement methods (P < 0.01). Significant differences were found between D2 and D8 for BIS R0 only (P < 0.01).

**Table 2:** **Comparison of the measured swelling according to the measurement method**

**Percentage difference with healthy side (mean SD)**			
	**Volume mean (SD)**	**Circumference mean (SD)**	**BIS R0 mean (SD)**
**Baseline**	3.0 (5.1)	2.6 (4.1)	3.5 (5.9)
**Day 2 post surgery**	14.7 (9.5)*	11.1 (5.7)*	29.9 (9.8)*
**Day 8 post surgery**	14.9 (8.2)	11.7 (4.1)	38.27 (7.8)^†^

*Significant difference with baseline P < 0.01.

^†^Significant difference with day 2 P < 0.01.

Legends = BIS R0: Bioimpedance spectroscopy; SD: Standard Deviation.

The ANOVA found a non-significant difference between the evaluators (P = 0.48) as well as between the first and the second measurement by the same evaluator (P = 0.54).

The ICC are reported in Table 3 for the relationship between the first and the second measurement of the same evaluator and for the relationship between the evaluators for their respective first and second measurements.

**Table 3:** **Intra- and inter- evaluator intraclass coefficient of correlation for BIS R0**

**Bioimpedance BIS R0 reliability for limb percentage difference**			
	**Baseline**	**D2**	**D8**
**Intra-evaluator ICC (1** ^**st**^ **; 2** ^**nd**^ **evaluator)**	0.90; 0.93	0.99; 0.96	0.98; 0.98
**Inter-evaluator ICC (1** ^**st**^ **; 2** ^**nd**^ **measurement)**	0.89; 0.97	0.98; 0.98	0.97; 0.99

All ICC are significant at P < 0.01.

Legends = BIS R0: Bioimpedance spectroscopy; ICC: Intra-class Correlation Coefficient; 1^st^: first; 2^nd^: second.

The Bland and Altman analysis for the difference between the evaluators showed that the bias and the limits of agreement were, respectively −0.3% (95% confidence interval (95% CI) -1.0 to 0.5) and −4.8 to 4.4 at baseline, −0.2% (95% CI −0.8 to 0.5) and −4.5 to 4.1 at D2, and −0.5% (95% CI −0.9 to 0.0) and −3.4 to 2.4 at D8. The inter-evaluator SEM were 0.6% at baseline, 0.3% at D2 and 0.2% at D8.

The Bland and Altman analysis for the difference between the first and second measurement of the identical evaluator showed that the bias and the limits of agreement were, respectively, −0.2% (95% CI −1.0 to 0.6) and −5.2 to 4.8 at baseline, −0.6% (95% CI −1.2 to 0.1) and −4.6 to 3.4 at D2, and −0.3% (95% CI −0.8 to 0.1) and −3.2 to 2.6 at D8. The intra-evaluator standard errors of measurement were 0.76% at baseline, 0.3% at D2 and 0.2% at D8.

The correlations between the measurement methods were all significant at baseline and D2. At D8, the volume - circumference correlation was significant, whereas the volume - BIS R0 (P = 0.13) and circumference - BIS R0 (P = 0.06) correlations were not significant (Table 4).

**Table 4:** **Correlation between the measurement methods**

**Spearman correlations between BIS R0, volume and circumference**			
	**R0 BIS-Volume**	**R0 BIS-Circumference**	**Volume-Circumference**
**Overall**	0.73*	0.75*	0.84*
**Baseline**	0.71*	0.56*	0.67*
**D2**	0.62*	0.57*	0.63*
**D8**	0.33 NS	0.39 NS	0.68*

*Significant at P < 0.01; NS non-significant.

Legends = BIS R0: Bioimpedance spectroscopy; D2: two days after total knee arthroplasty; D8: eight days after total knee arthroplasty.

The Cohen’s *d* for the effect size for the baseline - D2 difference was 3.32 for BIS R0, 1.49 for volume and 1.71 for circumference. It was 5.07, 1.76 and 2.22, respectively, for the baseline - D8 difference and 0.95, 0.08 and 0.11, respectively, for the D2 – D8 difference. The sensitivity, the specificity and the optimal threshold to discriminate the pre- from the post-surgical swelling states are presented in Table 5.

**Table 5:** **Comparison of the diagnostic power of the measurement methods**

**Diagnostic power comparison**					
**Stage**	**Variable**	**AUC [95% IC]**	**Threshold**	**Sensitivity (%)**	**Specificity (%)**
**D2**	**BIS R0**	0.99* [0.98 - 1.00]	13.4	0.96	0.96
	**Volume**	0.84* [0.72 - 0.96]	6.1	0.83	0.79
	**Circumference**	0.91* [0.81 - 1.00]	5.6	0.92	0.83
**D8**	**BIS R0**	1.00* [0.99 - 1.00]	13.8	1	0.96
	**Volume**	0.92* [0.84 - 1.00]	7.7	0.92	0.92
	**Circumference**	0.95* [0.90 - 1.00]	5.8	0.96	0.88

Legends = AUC: area under the curve; [95% IC]: 95% confidence interval; *: P < 0.01; BIS R0: Bioimpedance spectroscopy.

## Discussion

This study investigated the measurement properties of the BIS R0 method for the evaluation of swelling following primary TKA surgery. The population characteristics concerning the age and BMI are representative of the typical population of patients undergoing TKA surgery [44,45].

### Principal findings

At the baseline, slight swelling of comparable magnitude was detected by all of the measurement methods. This swelling was probably related to osteoarthritis of the knee, which was the reason for the surgical intervention. Conversely, the limb percentage difference at D2 and D8 differed considerably with the measurement method because BIS R0 measured a larger quantity of fluid than the concurrent methods. This discrepancy is the result of methods not measuring the same parameters. The volume and circumference measured include all of the anatomical structures. The responsiveness of these measurement methods is questionable because the swelling has only a marginal influence on the measured circumferences. As an example, the limb volume does not double when the quantity of extracellular fluids doubles. Conversely, the current at the R0 frequency flows only through the extracellular fluid (ECF) [36]. As BIS R0 measurement reflects solely the ECF, any extracellular fluid variation is directly accounted for, in measurement. Hence, variations in swelling, which is essentially within the extracellular space, produce larger changes in BIS R0 than in the volume or circumference [46].

No significant difference was found between the first and the second measurement of either of the evaluators, as well as between the measurements of the two evaluators. The intra- and inter-evaluator reliability was excellent following surgery (0.96 to 0.99). It was also excellent at baseline, with the exception of the first comparison of the evaluators’ measurements, which was good (ICC = 0.89). Consistent with these results, the Bland and Altman analysis showed a bias below 0.6% in all of the cases and a maximum absolute value of limits of agreement below 5.2%. The ICC values found in this study are comparable to those reported for the BIS ratio in upper limb lymphedema, as well as to those found for the volume determined by the truncated cone method or the circumference [28,47,48].

The margin of error when repeated measurements were performed by the same or several evaluators is limited. The clinical follow-up monitoring of swelling could be performed by several evaluators without significant influence on the outcome. Variations below 5.6% might be attributable to measurement error in the performance of individual measurements. This magnitude of limits of agreement could be considered adequate compared with the magnitude of post-surgical swelling, which reached 30% using BIS R0.

### Relation to the literature

The overall correlations of BIS R0 and volume (R = 0.73) and circumference (R = 0.75) were good to excellent [43]. Previous studies also found that the comparison of the raw impedance data between the affected limb vs. the unaffected limb was valid and correlated to volume [33,36,38,39,40]. The values observed in this study were within the range observed in previous studies that addressed the evaluation of upper arm lymphedema [38] or lower limb post-traumatic swelling [37,39]. Although post-surgical swelling has a less homogeneous composition than lymphedema, the evaluation of ECF appears to be of comparable validity for both conditions. The examinations of the correlations at definite stages highlight that the relationship between BIS R0 and other measurement methods was lost at D8. It is possible that the fluid electrolyte content evolved between D2 and D8 and influenced the BIS measurement. If this is the case, the BIS measurement of fluid content would be biased. An alternative hypothesis is that the post-surgical muscular atrophy altered the volume and circumference measurements. Consistent reduction of muscular mass has been observed at the early postsurgical stage following TKA [49]. This atrophy induces an underestimation of the postsurgical volume and circumference which could explain the larger progression of swelling between D2 and D8 showed using BIS. A comparison of BIS and imaging methods providing specific fluid measurement would be of use for investigating these hypotheses to determine the precise relationship between BIS and swelling for all of the post-surgical stages.

The discriminatory power between the pre- and post-surgical swelling states was better for BIS R0 than for volume and circumference, at all stages. The sensitivity and specificity of BIS R0 were excellent at D2 and D8 (96% -100%). BIS limb ratio had previously demonstrated high sensitivity and specificity for the detection of upper arm lymphedema [36,37,41]. The analysis performed in this study addressed the capacity of the methods to discriminate the baseline from the post-surgical swelling state of osteoarthritic patients. In considering these results, that the participants presented with a mean (SD) swelling of 3.5% (5.9%) at baseline must be accounted for. The capacity of the methods to discriminate pathological cases from subjects who have absolutely no swelling at all would have been probably even higher and the pathological threshold could have been set at a lower value.

### Strengths and weaknesses of the study

In agreement with observed differences between the stages and diagnostic sensitivity, the BIS R0 displayed a considerably better responsiveness. The variations of swelling are more clearly captured using BIS R0 than volume or circumference measurements. The use of BIS R0 in clinical trials might help limit type 2 errors because the potential treatment effects are more apparent.

Concurrent validity rather that gold standard validity was investigated in this study. Based on a pragmatic approach, BIS R0 measurement properties were compared to methods currently used in clinics. The comparison with advanced imaging techniques would have provided a more precise estimation of the BIS R0 relationship with swelling volume. However, it would not have allowed the comparison with the cheap, non-invasive and straightforward methods that are routinely applied in practice.

The relationship between BIS R0 and the concurrent methods is specific to the context of post-surgical TKA surgery because the swelling composition and type of surgery might potentially alter this relationship. Previous validation should be undertaken before application to other contexts. Conversely, it is likely that the excellent reproducibility and high responsiveness that were found are essentially related to the characteristics of the measurement method. These properties should be transferable to other situations addressing lower limb swelling.

### Implications for practice

Overall, BIS R0 demonstrated equivalent or better measurement properties than the concurrent methods in all of the cases. It presents theoretical advantages over circumference and volume, which are not specific measurements of interstitial fluid and are influenced by postsurgical amyotrophy. It also presents practical advantages because the measurement is straightforward and clean and could be performed under the post-surgical dressing.

Applications are possible to evaluate the effect of the surgical and early post-surgical procedures on the occurrence of swelling as well as the efficiency of rehabilitation treatment for the reduction of swelling. Bioimpedance measurement might be of interest to determine the presumed relationships between swelling and mobility, pain, quadriceps strength or gait alterations [9-12].

### Future research

Though the overall correlations of BIS R0 with volume and circumference were good to excellent, further research is needed to explain why the relationship between BIS R0 and other measurements methods fluctuate over time. In this study, bioimpedance was compared to a concurrent method routinely used in clinics. The future comparison with advances imagery techniques that allow the differentiation of edema and muscle volume could provide a precise estimation of the relationship between the edema volume and the BIS R0.

## Conclusions

This study investigated the measurement properties of BIS R0 for the evaluation of swelling following TKA primary surgery. This method provided straightforward measurements of limb swelling that could be applied at the bedside of the patient following TKA surgery. The correlation of BIS R0 with the percentage of limb difference determined by volume or circumference was globally high. It was non-significant when uniquely considering the relationship with volume or circumference at D8. Further research is needed to explain the reason that the relationship between BIS R0 and other measurements methods used in this study fluctuate over time. BIS R0 demonstrated excellent intra- and inter- evaluator reliability, small standard errors of measurement and clinically acceptable limits of agreement. The diagnostic sensitivity and the responsiveness of BIS R0 were better than that of the concurrent measurements methods used in this study. The use of BIS R0 is adapted for reliable and responsive monitoring of swelling monitoring in clinical conditions. It is an efficient method for post-surgical follow up at the patient’s bedside and for research investigating the causes, consequences and treatment of swelling following TKA surgery.

### Key points

- **Findings:** The measurement of BIS R0 is a straightforward, valid, reliable and responsive method for lower limb swelling following TKA surgery.
- **Implications:** It is an efficient and convenient method for post-surgical follow-up at the bedside of the patient. It may also be used for research investigating the causes, consequences and treatment of swelling following knee arthroplasty.
- **Caution:** Though the overall correlations of BIS R0 with volume and circumference were good to excellent, further research is needed to explain the fluctuation of the relationship between BIS R0, knee circumference and limb volume over time.
